# Performance of the Rowland Universal Dementia Assessment Scale for the Detection of Mild Cognitive Impairment and Dementia in a Diverse Cohort of Illiterate Persons From Rural Communities in Peru

**DOI:** 10.3389/fneur.2021.629325

**Published:** 2021-07-07

**Authors:** Nilton Custodio, Rosa Montesinos, Monica M. Diaz, Eder Herrera-Perez, Kristhy Chavez, Carlos Alva-Diaz, Willyams Reynoso-Guzman, Maritza Pintado-Caipa, José Cuenca, Carlos Gamboa, Serggio Lanata

**Affiliations:** ^1^Servicio de Neurología, Instituto Peruano de Neurociencias, Lima, Peru; ^2^Unidad de diagnóstico de deterioro cognitivo y prevención de demencia, Instituto Peruano de Neurociencias, Lima, Peru; ^3^Unidad de Investigación, Instituto Peruano de Neurociencias, Lima, Peru; ^4^Servicio de Rehabilitación, Instituto Peruano de Neurociencias, Lima, Peru; ^5^Department of Neurology, University of North Carolina at Chapel Hill, Chapel Hill, NC, United States; ^6^Unidad de epidemiología, ITS y VIH, Facultad de Salud Pública y Administración, Universidad Peruana Cayetano Heredia, Lima, Peru; ^7^Grupo de investigación Molident, Universidad San Ignacio de Loyola, Lima, Peru; ^8^Facultad de Ciencias de la Salud, Universidad Científica del Sur, Lima, Peru; ^9^Atlantic Fellow, Global Brain Health Institute, University of California, San Francisco, San Francisco, CA, United States; ^10^Servicio de Neuropsicología, Instituto Peruano de Neurociencias, Lima, Peru; ^11^Carrera de Psicología, Facultad de Ciencias de la Salud, Universidad Privada del Norte, Lima, Peru; ^12^Department of Neurology, University of California, San Francisco, San Francisco, CA, United States; ^13^Global Brain Health Institute, University of California, San Francisco, San Francisco, CA, United States

**Keywords:** cognition, dementia, education, literacy, mild cognitive impairment, RUDAS, cultural factors

## Abstract

**Background:** The accurate diagnosis of neurocognitive disorders in illiterate Peruvian populations is challenging, largely owing to scarcity of brief cognitive screening tools (BCST) validated in these diverse populations. The Peruvian version of the Rowland Universal Dementia Assessment Scale (RUDAS-PE) is a BCST that relies minimally on educational attainment and has shown good diagnostic accuracy in an urban illiterate population in Peru, yet its psychometric properties in illiterate populations in rural settings of the country have not been previously investigated.

**Objectives:** To establish the diagnostic accuracy of the RUDAS-PE compared to expert clinical diagnosis using the Clinical Dementia Rating (CDR) Scale in healthy and cognitively impaired illiterate persons living in two culturally and geographically distinct rural communities of Peru.

**Methods:** A cross-sectional, population-based study of residents ≥ 50 years of age living in the Peruvian rural communities of Santa Clotilde and Chuquibambilla. A total of 129 subjects (76 from Santa Clotilde and 53 from Chuquibambilla) were included in this study. Gold standard diagnostic neurocognitive evaluation was based on expert neurological history and examination and administration of the CDR. Receiver operating characteristics, areas under the curve (AUC), and logistic regression analyses were conducted to determine the performance of RUDAS-PE compared to expert gold standard diagnosis.

**Results:** Compared to gold standard diagnosis, the RUDAS-PE was better at correctly discriminating between MCI and dementia than discriminating between MCI and controls in both sites (97.0% vs. 76.2% correct classification in Chuquibambilla; 90.0% vs. 64.7% in Santa Clotilde). In Chuquibambilla, the area under the curve (AUC) of the RUDAS to discriminate between dementia and MCI was 99.4% (optimal cutoff at <18), whereas between MCI and controls it was 82.8% (optimal cutoff at <22). In Santa Clotilde, the area under the curve (AUC) of the RUDAS to discriminate between dementia and MCI was 99.1% (optimal cutoff at <17), whereas between MCI and controls it was 75.5% (optimal cutoff at <21).

**Conclusions:** The RUDAS-PE has acceptable psychometric properties and performed well in its ability to discriminate MCI and dementia in two cohorts of illiterate older adults from two distinct rural Peruvian communities.

## Introduction

The neurocognitive disorders, which include mild neurocognitive disorder (mild cognitive impairment, MCI) and major neurocognitive disorder (dementia) (1), are among the most prevalent age-related disorders worldwide and lead to substantial morbidity and mortality (2, 3). In Latin America, the prevalence of all forms of dementia is expected to rise from 7.8 million in 2013 to 27 million by the year 2050 (4, 5). Among the known modifiable risk factors for the neurocognitive disorders, low educational attainment appears to be both an independent risk factor for dementia (6) as well as a marker of poor prognosis, particularly in mid- to low-income regions such as Latin America (7–9).

In Peru, 6.0% of the population is illiterate and 20.0% of Peruvians who are able to receive formal education do not advance beyond elementary school (10). The highest illiteracy rates of the country are observed in rural populations, where up to 43.0% of adults report elementary school as their highest level of education (compared to 16.0% in urban settings) (11); among older adults (those ≥ 60 years of age) living in rural communities, 41.6% are illiterate (compared with 12.3% in urban settings) (11). Therefore, early, accurate diagnosis of neurocognitive disorders in rural Peruvian communities with low educational attainment represents a critical first step toward the proper allocation of resources for the care of these vulnerable older adults (12).

A significant barrier to the early, accurate diagnosis of older adults with neurocognitive disorders in rural Peruvian communities with low educational attainment relates to the fact that the majority of cognitive tests available to screen for cognitive impairment have been developed in relatively highly educated subjects from urban settings. Several popular brief cognitive screening tools (BCST) have been adapted and validated in Spanish-speaking illiterate or low literacy populations, including the Mini Mental State Exam (MMSE), Cognitive State Test (COST), Montreal Cognitive Assessment (MOCA), and the Memory Alteration Test (M@T). Yet, all of these BCSTs have important limitations, most notably their poor ability to discriminate between MCI and dementia cases, as well as their poor ability to help detect different etiologies (or types) of dementia (13–16).

The Rowland Universal Dementia Assessment Scale (RUDAS) was developed to address the limitations commonly encountered when using BCSTs to evaluate neurocognitive disorders in vulnerable populations. It is minimally influenced by educational attainment and measures a wide range of cognitive domains, hence it is promising for the detection of different types of dementia in sociodemographically diverse populations (17). Our group has validated the Peruvian version of the RUDAS (RUDAS-PE) in low literacy and illiterate populations in the capital city of Lima, Peru (18–20), where RUDAS-PE was useful in differentiating MCI from dementia with high sensitivity (89.0%) and specificity (93.0%) (21). In the present study we expand on this body of knowledge by testing the performance of the RUDAS-PE in two culturally distinct cohorts of illiterate subjects from two rural communities in Peru. We hypothesize that, owing to its low reliance on subject educational attainment, the RUDAS-PE will perform well in these study populations compared to gold standard diagnosis.

## Methods

### Participants

This study was approved by the ethics committee of the Institute of Tropical Medicine “Daniel Alcides Carrión” of the Universidad Nacional Mayor de San Marcos with approval number CIEI-2018-021. Participants and their study partners were asynchronously recruited from two geographically and culturally distinct rural communities of Peru: Santa Clotilde (January to September 2019) and Chuquibambilla (September 2019 to February 2020). Rurality was defined based on a population-density international classification system used by the World Bank (22). Santa Clotilde lies along the Napo River in the Peruvian Amazon region of the department of Loreto, near the border with Ecuador and Colombia; Chuquibambilla is located in the highlands of southern Peru in the district of Pangoa within the department of Junín. Santa Clotilde is located 10 h from the urban center of Iquitos and is accessible only by boat, whereas Chuquibambilla is located 1 h from the urban center of Satipoand is accessible by land.

In both sites, we recruited illiterate Spanish-speaking monolingual or bilingual (with ≥ 5 years of experience speaking Spanish) individuals who were ≥ 50 years of age at the time of enrollment. Illiteracy was determined using guidelines established by the Peruvian National Institute of Statistics: First, subjects were asked, *How many years of school did you attend?* Those who reported more than 1 year of formal education were excluded. Those who reported never attending school or completing <1 year of formal schooling were asked, *Are you able to read and write?* Those who reported being able to read and/or write were excluded.

We excluded individuals with history of: (1) physical limitations that might interfere with the neurocognitive evaluation, including hearing loss and uncorrected visual impairment [particularly problems with color discrimination, as assessed by the Dvorine color discrimination screening test (23)], (2) large vessel stroke, (3) developmental disabilities affecting cognitive performance, (4) neurocognitive deficits due to severe head trauma, (5) severe/poorly controlled psychiatric illness (depression, addiction disorder, bipolar disorder, schizophrenia, etc.), and (6) advanced neurocognitive impairment defined as severe compromise of activities of daily living regardless of etiology (i.e., stroke, Parkinson's Disease, traumatic brain injury), to the extent that the individual is fully dependent on caregivers and thus unable to participate in cognitive testing. In addition, we excluded individuals who reported taking any of the following medications within 7 nights prior to the assessment: opioid analgesics, decongestants, antispasmodics, anti-emetics, anti-cholinergics, anti-arrhythmics, anti-depressants, anti-psychotics, anti-anxiety and anti-epileptic medications such as valproate, phenobarbital, phenytoin, carbamazepine and levetiracetam.

All participants in this study were recruited from community health centers located in Santa Clotilde (one main clinic and three small satellite clinics) and Chuquibambilla (one main clinic). Recruitment occurred via simple random sampling in partnership with staff at each clinic. The outreach and recruitment process was as follows: (1) study approval was obtained from the directors of each of the community health centers; (2) primary care providers disseminated pertinent study information via existing communication streams (i.e., flyers, radio, face-to-face encounters between the patient and their provider); (3) individuals interested in participating in our study were flagged and the research team was informed of their upcoming clinical appointment dates; (4) the research team responsible for consenting and evaluating patients were present on site during the day of their scheduled clinical appointments; (4) participants were consented on site and were evaluated during the day of their clinical appointment. Attempts to minimize referral bias were made by random sampling of participants who presented to the clinic for their regular care.

All interested participants who met inclusion and exclusion criteria were consented verbally and fingerprints were used in lieu of signatures on the consent form. Each participant's family member or partner (spouse, close relative or friend) was also consented. Once consented and enrolled, each participant underwent an expert gold standard clinical diagnostic evaluation and administration of the RUDAS-PE by blinded team members as described below.

### Neurocognitive Measures

#### Clinical Diagnostic (Gold Standard) Evaluation

All participants included in this study underwent a gold standard diagnostic evaluation by trained and experienced health professionals from the “Instituto Peruano de Neurociencias” (IPN), located in Lima, Peru, which specializes in the care and clinical research of persons with neurocognitive disorders. The evaluating team included neuropsychologists [JC, CG], a neurologist with sub-specialty training in dementia [NC], and a neuro-rehabilitation specialist with experience in the evaluation and management of patients with dementia [RM]. These health professionals have formal training and experience in the administration and interpretation of various BCSTs and the Clinical Dementia Rating (CDR) scale. Given that, to our knowledge, this is the first neurocognitive health study conducted in these rural Peruvian communities and therefore there is no validated gold standard of diagnosis, for this study the diagnostic gold standard was based on an expert neurological examination and results from the CDR scale (24).

The CDR is a well-validated, semi-structured interview of the individual and a reliable informant or collateral source (e.g., family member, close relative or friend) used to characterize six domains of cognitive and functional performance: memory, orientation, judgment and problem solving, community affairs, home and hobbies, and personal care (24). Among its advantages, the CDR offers a global clinical assessment independently from other psychometric tests, does not require a baseline assessment, and has good inter-evaluation reliability, concurrent validity, and predictive validity; among its relative disadvantages, it takes approximately 30 min to administer and requires experienced clinical judgement in order to accurately obtain pertinent information (24–26).

In accordance with published research studies showing that the CDR can accurately distinguish MCI from dementia (25), for this study we assigned subjects with a CDR = 0 to the “control” group, subjects with CDR = 0.5 to the “MCI” group (26), and subjects with CDR = 1 or 2 to the “dementia” group. Cases in which the CDR was unclear were resolved via a multidisciplinary consensus meeting.

#### Peruvian Version of the Rowland Universal Dementia Assessment Scale (RUDAS-PE)

The RUDAS-PE takes approximately 10 min to administer and includes six cognitive domains, starting with immediate memory registration, followed by visuospatial orientation, motor praxis, visuospatial construction, judgment, recent episodic memory and language. It produces a maximum score of 30, where a low score denotes poor cognitive performance. The Spanish version of the RUDAS-PE can be found as a Supplementary Material to this article.

The study personnel who administered the RUDAS-PE were a resident physician in geriatrics from *Universidad Peruana Cayetano Heredia (UPCH)* Medical School [KC] and a certified practicing neurologist [WR], both of whom who had been previously trained in administering the RUDAS-PE and other BCSTs following previously reported procedures (21) as part of their training experience at the IPN. These evaluators (KC and WR) were blinded to the results of the gold standard clinical diagnostic evaluation.

### Statistical Analyses

Following the assignment of the study groups (control, MCI and dementia), the performance scores on the RUDAS-PE of individuals within each group were reviewed in order to carry out statistical analyses of the cohort demographic characteristics and the psychometric properties of the RUDAS-PE. The 95% confidence intervals were calculated, and significance level was set at 0.05. All analyses were completed using STATA software (version 12.0).

Descriptive statistics were obtained from cohorts in each of the two study sites (Chuquibambilla and Santa Clotilde), and compared by pairing cognitive groups with one another (controls, MCI and dementia). *T*-tests (for discrete variables) and Chi Square (for categorical variables) were then calculated.

RUDAS-PE scores and relationships between demographic and clinical characteristics (age, sex, city access, occupation, hypertension, diabetes, myocardial infarction, sedentary lifestyle and smoking) were modeled by adjusting polynomial curves to generate estimated means. We used a second-degree model that suppressed covariate centering, and variables were selected using backwards stepwise regression (backwards elimination) using a significance level of 0.05. Standardized beta coefficients (β) were reported to describe the strength of the association between each predictor and the RUDAS-PE score.

Internal consistency was evaluated using Cronbach's alpha coefficient. Sequential cognitive domains were removed from the RUDAS-PE to evaluate coefficient changes. Convergent validity was evaluated using Spearman correlation coefficient comparisons between RUDAS-PE total scores and scores on individual cognitive domains compared with CDR total scores. Logistic regression (logit) was performed for each pair of study groups (dementia/MCI, MCI/control and dementia/control) using a two-variable model with the final diagnosis as the dependent variable and the RUDAS-PE as the independent variable. Discriminant validity was determined by measuring the average total score of the RUDAS-PE and each of its domains within the three groups (controls, MCI and dementia). These were compared using means of independent samples *t*-test, calculation of the area under the curve (AUC) and Receiver Operating Characteristics (ROC) curves. In addition, we determined the percentage of correctly classified individuals and calculated multivariate analyses of variance (MANOVA). Diagnostic accuracy was evaluated by *post-hoc* analysis means used to calculate ROC curves and ROC plots. These were adjusted according to certain cut-off points allowing calculation of AUC values. In addition to calculating diagnostic accuracy, sensitivity, specificity, positive predictive values, negative predictive values, positive likelihood ratios (LR+) and negative likelihood ratios (LR) were calculated for different cut-off points for the RUDAS-PE in both regions. The maximum values of these measurements were the selection of the sensitivity, specificity and predictive value cut-off points.

## Results

### Demographics and Descriptive Statistics

A total of 129 subjects (53 from Chuquibambilla, 76 from Santa Clotilde) were included in this study Nearly 40.0% were female with similar proportions across the three groups (controls, MCI, dementia) in both sites (Table 1). The mean age of the participants from Chuquibambilla was 69.6 ± 5.3 years and 70.9 ± 5.4 years in Santa Clotilde. The MCI group was younger than the dementia group in both sites, but there were no statistically significant age differences between the control and MCI group at both sites.

**Table 1:** Demographic characteristics and brief cognitive test performance by study group in Chuquibambilla, Junín, and Santa Clotilde, Loreto. 2019.

	**Chuquibambilla**						**Santa Clotilde**					
	**Control (*n* = 20)**	**MCI (*n* = 22)**	**Dem (*n* = 11)**	***P*-value Control vs. MCI**	***P*-value MCI vs. Dem**	***P*-value Control vs. Dem**	**Control (*n* = 16)**	**MCI (*n* = 18)**	**Dem (*n* = 42)**	***P*-value Control vs. MCI**	***P*-value MCI vs. Dem**	***P*-value Control vs. Dem**
Female (%)	10 (50.0)	12 (52.4)	6 (54.5)	0.51	0.64	0.55	7 (43.7)	10 (55.6)	6 (50.0)	0.37	0.53	0.52
Age in years, mean (SD)	69.2 (6.5)	67.6 (4.7)	71.9 (4.6)	0.60	**0.00**	0.09	71.3 (7.5)	67.6 (3.3)	73.8 (5.5)	0.16	**0.00**	0.31
RUDAS-PE score, mean (SD)	23.0 (1.9)	20.8 (1.6)	14.9 (1.8)	**0.00**	**0.00**	**0.00**	22.63 (2.68)	20.72 (1.74)	15.17 (1.64)	**0.01**	**0.00**	**0.00**

*MCI, mild cognitive impairment; Dem, Dementia; SD, standard deviation; RUDAS-PE, rowland universal dementia assessment scale, Peruvian version. Bold values mean p < 0.05*.

Dementia groups at both sites demonstrated lower performance on the RUDAS-PE compared to the other groups (MCI and controls); moreover, participants with MCI performed worse than controls at both sites. The participants with dementia in the Chuquibambilla site performed worse on the RUDAS-PE compared to participants with MCI, with controls performing the best of all groups. In Santa Clotilde, a similar pattern was observed in RUDAS-PE scores across groups (Table 1). No statistically significant differences were found between the two study sites.

**Figure 1:**
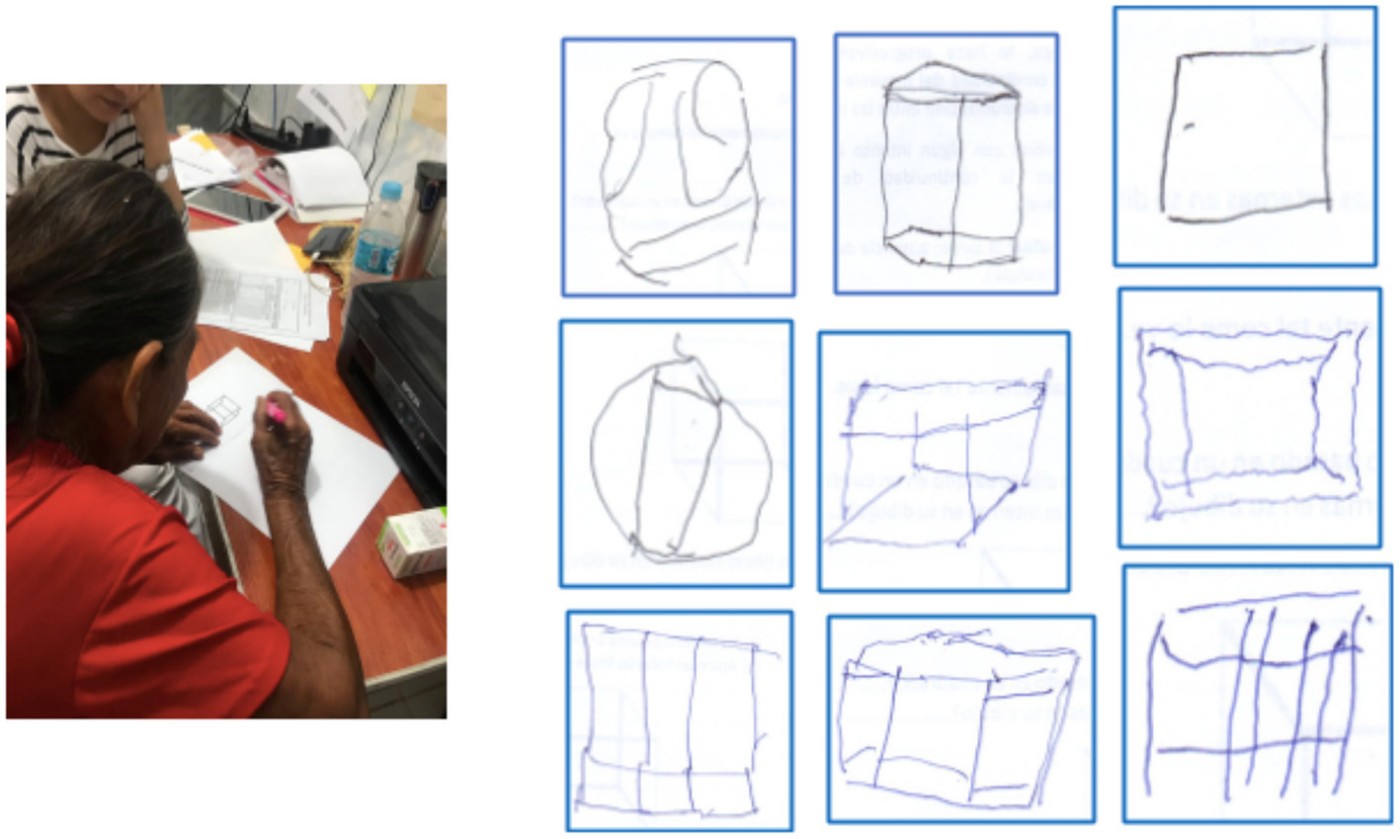
Examples of common errors in drawing the cube in the visuo-spatial construction domain in illiterate individuals from the Santa Clotilde. Note absence of three-dimensionality and distorted angles.

We performed an additional analysis to test performance on the RUDAS-PE per neurocognitive domain in MCI and control groups of illiterate persons in rural settings compared with illiterate persons in urban settings (from Ventanilla, in Lima, Peru), and found that rural illiterate individuals performed worse in motor praxis and visuospatial construction (Table 2). Figure 1 shows examples of the types of difficulties most residents of Santa Clotilde demonstrated when asked to copy the cube. Conversely, the sample of participants with MCI and controls from Ventanilla performed significantly worse than the sample from Chuquibambilla within the memory domain (Table 2).

**Table 2:** Performance on the Peruvian version of the Rowland Universal Dementia Assessment Scale (RUDAS-PE) by cognitive domain among control and MCI groups from rural and urban illiterate Peruvian communities.

**Rudas, cognitive domains**	**Chuquibambilla control + MCI (*n* = 42)**	**Santa Clotilde control + MCI (*n* = 34)**	**Ventanilla control + MCI (*n* = 124)**	***P*-value chuquibambilla vs. ventanilla**	***P*-value Santa Clotilde vs. ventanilla**
Visuo-spatial orientation	4.3 (0.8)	4.4 (0.8)	4.4 (0.5)	0.42	0.66
Praxis	1.0 (0.7)	1.0 (0.8)	1.6 (0.5)	**0.00**	**0.00**
Visuospatial construction	0.2 (0.4)	0.2 (0.4)	0.7 (0.6)	**0.00**	**0.00**
Judgment	1.8 (0.9)	1.7 (1.1)	1.9 (0.8)	0.38	0.28
Memory	7.0 (1.2)	6.7 (1.2)	6.3 (1.3)	**0.00**	0.09
Language	7.5 (0.7)	7.6 (0.7)	7.3 (0.8)	0.09	0.10

*MCI, mild cognitive impairment; SD, standard deviation. Data presented as mean (SD). Bold values mean p < 0.05*.

### Internal Consistency

The Cronbach's alpha coefficient for RUDAS-PE among rural illiterate older adults from both sites was 0.6 (0.5 in Chuquibambilla and 0.6 in Santa Clotilde). The internal consistency by study group in Chuquibambilla was 0.5 in the control group, 0.6 in MCI group and 0.5 in the dementia group. In the Santa Clotilde cohort, the Cronbach's alpha coefficient was 0.5 in the control group, 0.4 in the MCI group and 0.6 in the dementia group. When a cognitive domain evaluated in the RUDAS-PE was sequentially removed, the overall Cronbach's alpha coefficient did not increase, and the value decreased instead. For this reason, all domains contributed positively to the RUDAS-PE and were shown to be consistent within the test.

### Construct Validity

Spearman correlations were obtained between RUDAS-PE compared with CDR scores. The correlation between RUDAS-PE and CDR scores was 0.9 (SD 0.03; 95% CI) for the cohort [0.9 (SD 0.06; 95% CI) for Chuquibambilla and 0.9 (SD 0.1; 95% CI)] for Santa Clotilde.

### Discriminant Validity

**Figure 2:**
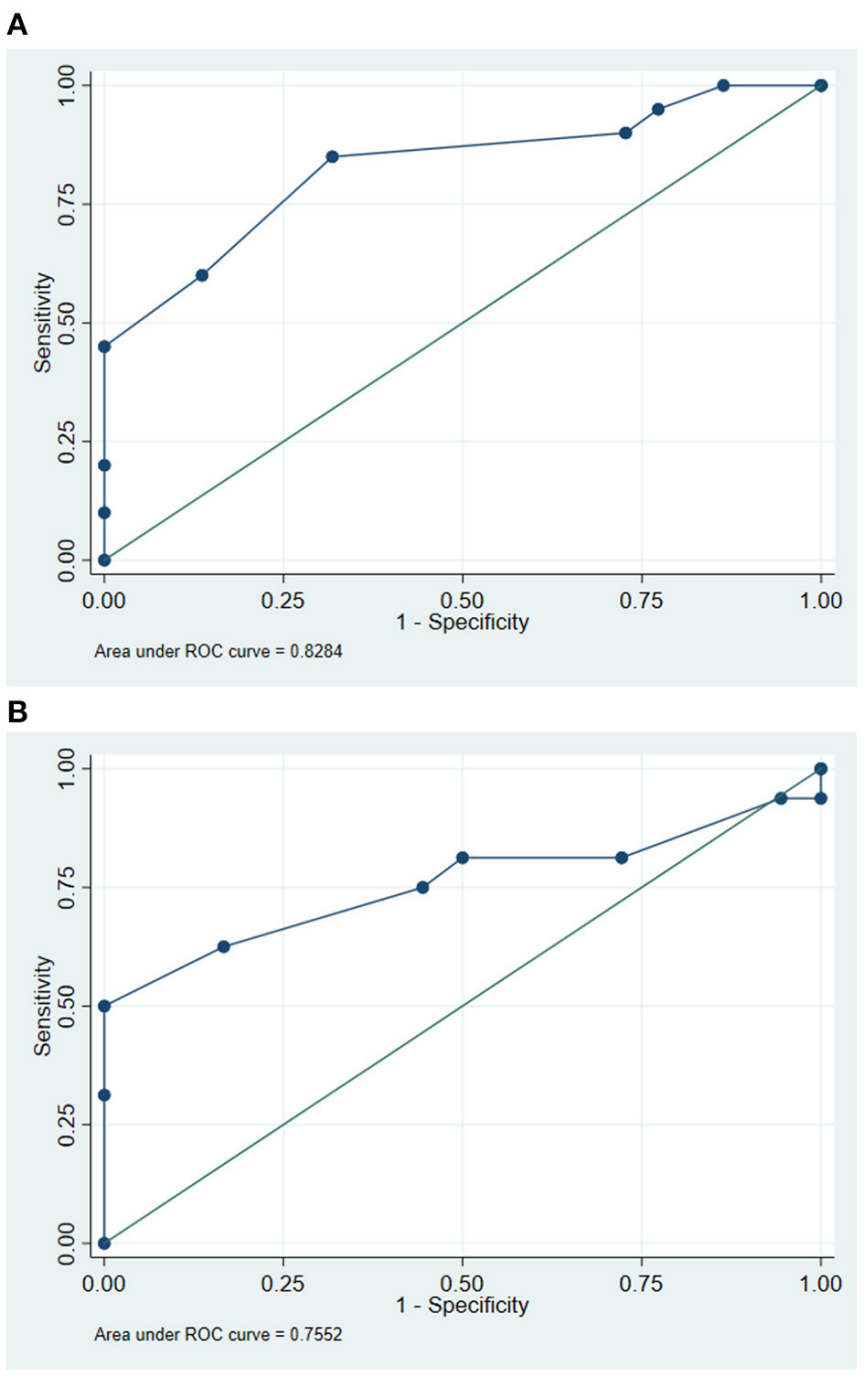
Receiver-operating characteristic (ROC) curve for the Peruvian version of the Rowland Universal Dementia Assessment Scale (RUDAS-PE) for discrimination between mild cognitive impairment (MCI) and control groups. Chuquibambilla, Junín. **(B)** Receiver-operating characteristic (ROC) curve for the Peruvian version of the Rowland Universal Dementia Assessment Scale (RUDAS-PE) for discrimination between mild cognitive impairment (MCI) and control groups. Santa Clotilde, Loreto.

**Figure 3:**
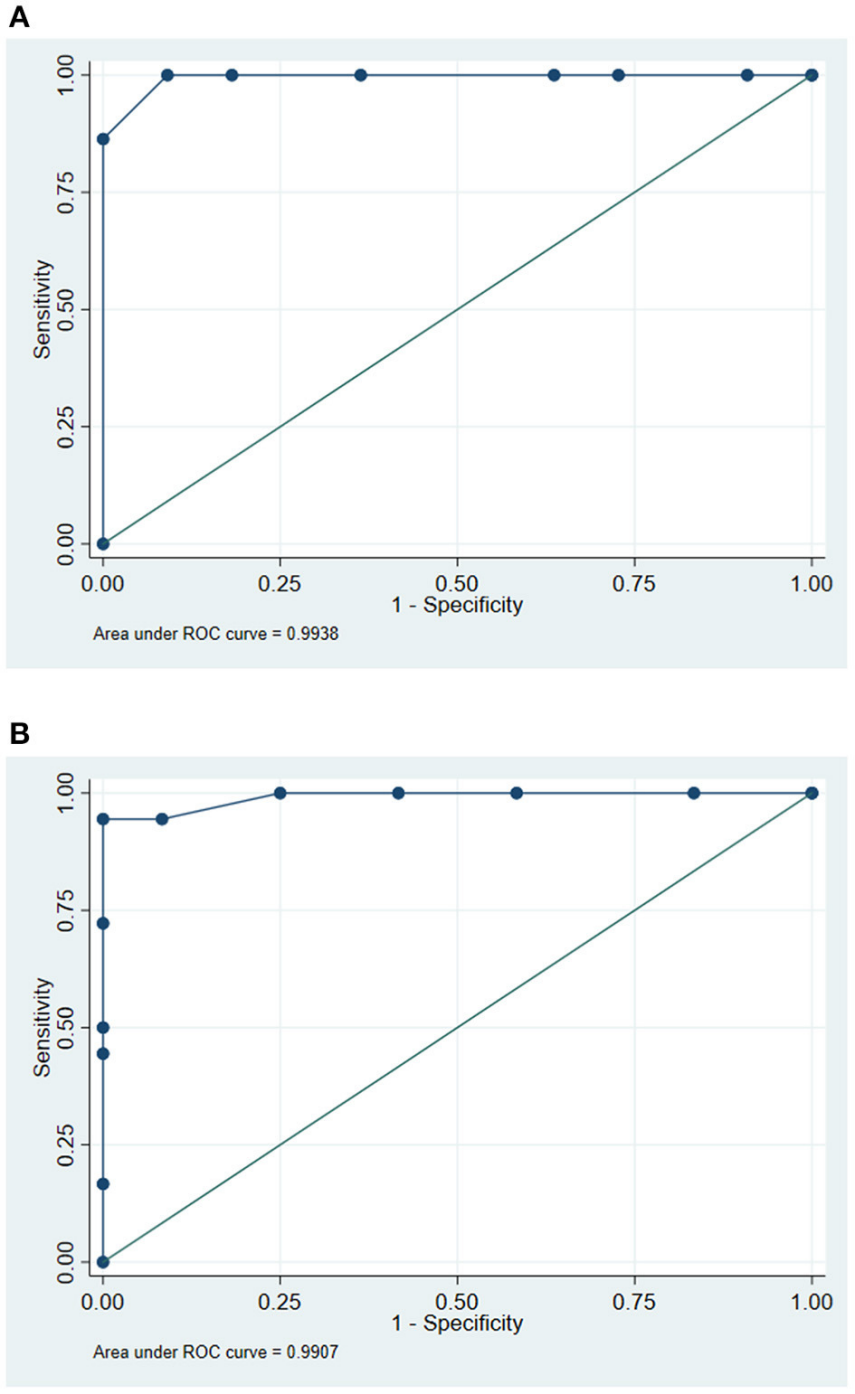
Receiver-operating characteristic (ROC) curve for the Peruvian version of the Rowland Universal Dementia Assessment Scale (RUDAS-PE) for discrimination between mild cognitive impairment (MCI) and dementia groups. Chuquibambilla, Junín. **(B)** Receiver-operating characteristic (ROC) curve for the Peruvian version of the Rowland Universal Dementia Assessment Scale (RUDAS-PE) for discrimination between mild cognitive impairment (MCI) and dementia groups. Santa Clotilde, Loreto.

For each study site, the ROC curve and an AUC for the RUDAS-PE were calculated for the following three study group comparisons: (1) control vs. MCI, (2) control vs. dementia, and (3) MCI vs. dementia. Figure 2A shows the RUDAS-PE ROC curve (AUC=0.82) to discriminate between MCI and controls in Chuquibambilla. Figure 2B shows the RUDAS-PE ROC curve (AUC = 0.75) to discriminate between MCI and controls in Santa Clotilde. Figures 3A,B show the RUDAS-PE ROC curves for Chuquibambilla and Santa Clotilde to discriminate MCI and dementia, with AUCs of 0.99 and 0.99, respectively.

### Diagnostic Accuracy

In the Chuquibambilla site, the optimal cut-off score on the RUDAS-PE to differentiate controls from the MCI group was 22 (sensitivity of 85.0%, specificity of 68.2%), with a high proportion of false positives (24.0%); the optimal cut-off score to differentiate participants with MCI from those with dementia was 18 (sensitivity of 100.0%, specificity of 90.9%) with a low proportion of false positives (3.0%). In the Santa Clotilde group, the optimal cut-off score on the RUDAS-PE to differentiate controls from MCI was 21 (sensitivity of 81.3%, specificity of 50.0%), but with a high proportion of false positives (35.0%); the optimal cut-off score to discriminate between MCI and dementia on the was 17 (sensitivity of 100.0%, specificity of 75.0%) with a 10.0% false positive rate (Table 3).

**Table 3:** Cut-off points and diagnostic performance for the Peruvian version of the Rowland Universal Dementia Assessment Scale (RUDAS-PE) to discriminate between controls and participants with mild cognitive impairment (MCI) and dementia in Chuquibambilla, Junín and Santa Clotilde, Loreto.

**Diagnostic performance**	**Discrimination between controls and participants with MCI**		**Discrimination between participants with MCI and dementia**	
	**Chuquibambilla**	**Santa Clotilde**	**Chuquibambilla**	**Santa Clotilde**
Optimal cutoff point	22	21	18	17
Sensitivity, %	85.0	81.3	100.0	100.0
Specificity, %	68.2	50.0	91.0	75.0
Correctly classified, %	76.1	64.7	97.0	90.0
Likelihood ratio +	2.67	1.63	11.00	4.00
Likelihood ratio –	0.22	0.38	0.00	0.00
AUC (95% CI)	0.83 (0.81–0.84)	0.76 (0.74–0.78)	0.99 (0.98–1.00)	0.99 (0.98–1.00)

*MCI, mild cognitive impairment; AUC, area under the curve; CI, confidence interval*.

## Discussion

To our knowledge, this is the first community-based study of the RUDAS-PE in illiterate persons living in rural settings of Peru, and also the first such study that tests the performance of RUDAS-PE in distinguishing between MCI and dementia. Our study results point to three main findings: (1) compared to expert clinical diagnosis, the RUDAS-PE was superior at discriminating between persons with MCI and persons with dementia than discriminating between persons with normal cognition and persons with MCI; (2) in both analyses (controls vs. MCI and MCI vs. dementia), the discriminatory abilities of the RUDAS-PE tended to be relatively weaker in the rural community of Santa Clotilde compared to the discriminatory abilities observed in the rural community of Chuquibambilla, thus suggesting that sociocultural factors unique to each community may influence cognitive testing performance among illiterate persons; and (3) compared to illiterate persons living in the highly urban Peruvian capital city of Lima, illiterate persons living in both rural communities in our study performed significantly worse on the domains of motor praxis and visuospatial construction on the RUDAS-PE, suggesting that unspecified sociocultural factors present in urban environments may influence performance on the RUDAS-PE in illiterate persons.

The first RUDAS validation study for Spanish-speakers was carried out among a sample of low literacy subjects in Santiago de Compostela, La Coruña, Spain (20) for an optimal dementia cut-off point of 21/22 (sensitivity of 94.9%, specificity of 75.0%). The same authors also performed a comparative analysis between the MMSE and RUDAS for dementia screening in a different Spanish cohort with low literacy and found that the best cut-off point for the RUDAS was 21/22 (sensitivity of 94.3%, specificity of 72.6%) (19). These results are similar to those obtained in our cohort from Chuquibambilla, with a cut-off point of 22 (sensitivity of 85.0%, specificity of 68.2%), but the values were lower for the cohort from Santa Clotilde, with a cut-off point of 21 (sensitivity of 81.3%, specificity of 50.0 and 64.7% of correctly classified) for a cut-off point of 21. This regional difference in the performance of the RUDAS-PE is noteworthy because it suggests the existence of yet unspecified environmental and cultural factors that may influence performance on the RUDAS-PE among illiterate persons. Indeed, the Santa Clotilde community is more geographically isolated that the Chuquibambilla community and is therefore considered to be “more rural”; moreover, these two communities embody their own distinct cultural practices, which may have also directly impacted performance on the RUDAS-PE in subjects from both communities. We believe these findings shed light on the importance of conducting further studies seeking to understand the potentially significant influence that sociocultural factors, even those at play within a country, have on cognitive testing performance.

It is important for all health professionals, especially those caring for vulnerable populations, to diagnose persons with neurodegenerative diseases early in their illness (mild neurocognitive disorder, or MCI) rather than late (major neurocognitive disorder, or dementia). Indeed, early diagnosis of neurodegenerative disease leads to better patient outcomes, including early initiation of pharmacologic and non-pharmacologic symptomatic therapies, early management of key modifiable risk factors, as well as early family education and planning for long-term care (27). In this regard, in our study, the RUDAS-PE showed promise in discriminating between controls and MCI, with the cut-off score in Santa Clotilde being one point lower than in Chuquibambilla (21 and 22, respectively). Of note, both cut-off scores were lower than the RUDAS-PE cut-off score obtained in our previously reported findings in an urban illiterate population in Lima, Peru (23) (21). Similarly, the specificity, percentage of correctly classified groups and the AUC for the RUDAS-PE was lower in the Santa Clotilde cohort (S = 50.0%; CC = 64.7%; AUC = 0.75) compared to Chuquibambilla (sensitivity [S] = 68.2%: correctly classified [CC] = 76.2% AUC = 0.8284). In turn, these were lower compared to those obtained among urban illiterate persons of Lima (S = 93.3%: CC = 91.1%; AUC = 0.98) (21). These results are difficult to compare with previously published studies, since most of them compare controls with persons with dementia, not MCI, and have been conducted in urban, higher-income populations; but again, these findings suggest that there are important cultural and environment factors to consider when assessing illiterate populations with mild cognitive changes in rural communities of Peru.

It is important to highlight that the screening utility of different BCST is dependent on the expected baseline rates of cognitive impairment in the population being sampled. Our study results suggest that a cut-off point of 21/22 is optimal for MCI screening and a cut-off point of 17/18 is optimal for dementia screening when the RUDAS-PE is used in illiterate persons form rural communities where the expected baseline rates of MCI and dementia are 17.7% and 32.3%, respectively (28). Whereas cut-off points of 23 and 19 may be optimal for predicting the presence or absence of MCI and dementia, respectively, in illiterate persons living in the city of Lima, where expected baseline rates of cognitive impairment differ (21, 29).

We found that compared to illiterate persons from the city of Lima our study subjects performed significantly worse on the domains of motor praxis and visuospatial construction on the RUDAS-PE. Previous studies have shown that low educational attainment is associated with poor performance in alternating hand movements and cube copying (30, 31). Similarly, many assessments of praxis, such as buccofacial movements, fine alternating movements of fingers, imitation of non-sensical movements, coordinated movement of both hands, line cancellation, and motor impersistence tasks tend to be more challenging for illiterate persons compared to highly educated professionals (32, 33). Furthermore, previous studies have shown that illiterate persons struggle with cube-drawing and reproduction of three-dimensional figures (34). Compared to highly educated persons, illiterate persons have been show to struggle to copy figures (cube, house, intersecting pentagons, complex Rey-Osterrieth figure), recognize superimposed figures, interpret a map, and draw a floor plan of a room (35). Thus, illiterate persons may be at a clinically significant disadvantage when undergoing cognitive testing, beyond that which would be expected based merely on their reading and writing skills. Several hypotheses have been postulated to explain this observation, including: (1) unfamiliarity with both the content and the test procedure itself (“testwiseness”), given that neuropsychological test formats are reminiscent of a school assignment rather than real-life activities; (2) lack of school-acquired strategies (explicit or implicit) for organizing and retaining information such as problem-solving skills, concentration, accurate expression of knowledge within an allotted time, and being internally motivated to perform well on tests; and (3) the potential effects of longstanding socioeconomic poverty in brain development. Regarding the latter point, it has been proposed that early deprivation of basic health and welfare needs (such as housing, nutrition, and health care) leads to chronic stress, dysfunction of the. hypothalamic-pituitary-adrenal axis, eventually leading to impairments in brain development and functioning (33). Our study findings add to this overall body of literature, but also introduce the possibility that the observed differences in neuropsychological testing performance observed in illiterate persons may be modified by unidentified “urban” factors, such as greater exposure to, and familiarity with, three-dimensional visual stimuli as a result of living in large cities.

Our study has important limitations. First, it was not powered to obtain more precise estimates of validity parameters and decrease the tendency of misclassifications. Second, there is a risk of random variation in participation rates despite attempts to minimize participation bias across the two study sites, which may have led to different reported rates of cognitive impairment between the two groups; however, the primary objective of our study was to determine the feasibility and validity of the RUDAS-PE in illiterate persons and not to establish the prevalence of cognitive impairment in these Peruvian communities. Third, given the cross-sectional design of the study (i.e. no longitudinal ascertainment of cognition) and lack of validated neuropsychological tests for Peruvian illiterate persons from rural settings, there is a probable tendency toward erroneous classifications of our study groups (controls, MCI and dementia); however, to compensate for this weakness we relied on previously trained and experienced clinicians to ascertain our study groups using expert neurological diagnosis and the CDR, which has been previously shown to accurately distinguish healthy controls, from MCI and dementia (25). Furthermore, the evaluating clinicians were blinded to the results of the RUDAS-PE. This procedure was followed to prevent incorporation of biases and overestimation of diagnostic accuracy for the RUDAS-PE assessments. Still, clinical diagnosis may be less accurate than when standardized neuropsychological assessment are included, as well as brain imaging. On the other hand, it should be noted that primary care physicians and geriatricians in Peru usually do not utilize comprehensive neuropsychological testing or neuroimaging when evaluating older adults with cognitive impairment, procedures that are usually performed only at specialized memory centers and academic institutions. Therefore, our study design is more reflective of the approach used in primary care when evaluating illiterate persons with cognitive impairment, and our study results suggest that the RUDAS-PE may be a good, briefer alternative to the GDS. Fourth, participants included in this study were from rural Peruvian communities that were fluent in Spanish and did not include Peruvians who speak native languages such as Quechua or Aymara. Therefore, the results or our study are reflective of the performance of the RUDAS-PE in Spanish-speaking illiterate rural Peruvians, and further studies are needed to determine influence of indigenous culture and bilingualism on the RUDAS-PE.

## Conclusions

We present the first study assessing the neurocognitive health of rural illiterate persons living in two culturally and geographically distinct regions of Peru. The RUDAS-PE was found to adequately distinguish MCI from dementia in our study. Diagnosing persons with neurodegenerative disease early in their illness (i.e., in MCI stages) is crucial in order to implement supportive therapies in a timely manner, intervene on modifiable risk factors for dementia early on in the disease course, and counsel family members on expectations of disease course promptly. Our study results suggest the existence of cultural and environmental factors that may influence performance on the RUDAS-PE among illiterate persons. Indeed, we believe it's important for researchers to account for the significant cultural and environmental diversity that exists within Latin America, even within Latin American countries, when developing and testing BCSTs in vulnerable populations in the region. Finally, we found that our study participants performed worse in motor praxis (alternating hand movements) and visuospatial construction (copy of the cube drawing), compared to the performance of urban illiterate individuals in Lima, Peru. These results further emphasizes the existence of “urban” factors other than educational level, such as test-taking familiarity, lack of school-acquired problem-solving skills and the potential role of chronic poverty on brain development, all of which influence the testing performance of illiterate persons from rural settings.

## Data Availability Statement

The original contributions presented in the study are included in the article/Supplementary Material, further inquiries can be directed to the corresponding author/s.

## Ethics Statement

The studies involving human participants were reviewed and approved by Institute of Tropical Medicine Daniel Alcides Carrión of the Universidad Nacional Mayor de San Marcos. The patients/participants provided their written informed consent to participate in this study.

## Author Contributions

All authors listed have made a substantial, direct and intellectual contribution to the work, and approved it for publication.

## Conflict of Interest

The authors declare that the research was conducted in the absence of any commercial or financial relationships that could be construed as a potential conflict of interest.
